# Supplementary material for: Effect of Androgen-Deprivation Therapy on Bone Mineral Density in Patients with Prostate Cancer: A Systematic Review and Meta-Analysis
Source: J Clin Med. 2019 Jan 18;8(1):113. doi: 10.3390/jcm8010113 (PMC6352073; doi:10.3390/jcm8010113)
Supplement: Supplementary file 1 [file jcm-08-00113-s001.pdf]

File S1. Search terms

PUBMED

("prostatic neoplasms"[MeSH Terms] OR ("prostatic"[All Fields] AND "neoplasms"[All Fields]) OR "prostatic neoplasms"[All Fields] OR ("prostate"[All Fields] AND "cancer"[All Fields]) OR "prostate cancer"[All Fields]) AND ((("androgens"[Pharmacological Action] OR "androgens"[MeSH Terms] OR "androgens"[All Fields] OR "androgen"[All Fields]) AND deprivation[All Fields]) OR (("androgens"[Pharmacological Action] OR "androgens"[MeSH Terms] OR "androgens"[All Fields] OR "androgen"[All Fields]) AND suppression[All Fields]) OR ("hormones"[Pharmacological Action] OR "hormones"[MeSH Terms] OR "hormones"[All Fields] OR "hormone"[All Fields]) OR ("gonadotropins"[MeSH Terms] OR "gonadotropins"[All Fields] OR "gonadotropin"[All Fields])) AND (("bone density"[MeSH Terms] OR ("bone"[All Fields] AND "density"[All Fields]) OR "bone density"[All Fields] OR ("bone"[All Fields] AND "mineral"[All Fields] AND "density"[All Fields]) OR "bone mineral density"[All Fields]) OR ("bone diseases, metabolic"[MeSH Terms] OR ("bone"[All Fields] AND "diseases"[All Fields] AND "metabolic"[All Fields]) OR "metabolic bone diseases"[All Fields] OR ("bone"[All Fields] AND "loss"[All Fields]) OR "bone loss"[All Fields]) OR ("bone density"[MeSH Terms] OR ("bone"[All Fields] AND "density"[All Fields]) OR "bone density"[All Fields]) OR (skeletal[All Fields] AND ("Change"[Journal] OR "change"[All Fields])) OR ("osteoporosis, postmenopausal"[MeSH Terms] OR ("osteoporosis"[All Fields] AND "postmenopausal"[All Fields]) OR "postmenopausal osteoporosis"[All Fields] OR "osteoporosis"[All Fields] OR "osteoporosis"[MeSH Terms]))

EMBASE

('prostate cancer'/exp OR 'prostate cancer') AND ('androgen deprivation'/exp OR 'androgen deprivation' OR 'androgen suppression therapy'/exp OR 'androgen suppression therapy' OR 'hormone'/exp OR 'hormone' OR 'gonadotropin'/exp OR 'gonadotropin') AND ('bone mineral density'/exp OR 'bone mineral density' OR 'bone loss or bone density' OR 'skeletal change or osteoporosis')

File S2. Excluded Studies

1. Alibhai, S.M.H.; Breunis, H.; Timilshina, N.; Hamidi, M.S.; Cheung, A.M.; Tomlinson, G.A.; Manokumar, T.; Samadi, O.; Sandoval, J.; Durbano, S. et al. Improving bone health in men with prostate cancer receiving androgen deprivation therapy: Results of a randomized phase 2 trial. Cancer 2018, 124, 1132-1140.
2. Alibhai, S.M.H.; Mohamedali, H.Z.; Panju, A.H.; Timilshina, N.; Breunis, H.; Canning, S.D.; Fleshner, N.E.; Krahn, M.; Naglie, G.; Tannock, I. et al. Impact of androgen deprivation therapy (adt) on bone mineral density (bmd) over 3 years in men with nonmetastatic prostate cancer. J. Clin. Oncol. 2012, 30.
3. Almatari, M.; Ali, P.; Willshaw, P.; El-Sharkawi, S. Impact on bone density of neoadjuvant hormone therapy for cancer prostate patients. Osteoporos Int. 2010, 21, S491.
4. Bernat, M.M.; Pasini, J.; Marekovic, Z. Changes in bone mineral density in patients with prostate cancer treated with androgen deprivation therapy. Coll. Antropol. 2005, 29, 589-591.
5. Berruti, A.; Dogliotti, L.; Terrone, C.; Cerutti, S.; Isaia, G.; Tarabuzzi, R.; Reimondo, G.; Mari, M.; Ardissone, P.; De Luca, S. et al. Changes in bone mineral density, lean body mass and fat content as measured by dual energy x-ray absorptiometry in patients with prostate cancer without apparent bone metastases given androgen deprivation therapy. J. Urol 2002, 167, 2361-2367; discussion 2367.
6. Daniell, H.W.; Dunn, S.R.; Ferguson, D.W.; Lomas, G.; Niazi, Z.; Stratte, P.T. Progressive

osteoporosis during androgen deprivation therapy for prostate cancer. J. Urol 2000, 163, 181-186.
7. Deng, J.H.; Yang, L.P.; Wang, L.S.; Zhou, D.F. Effect of androgen deprivation therapy on bone mineral density in prostate cancer patients. Asian J. Androl. 2004, 6, 75-77.
8. Furuta, A.; Yanada, S.; Takizawa, A.; Iwamuro, S.; Suzuki, M.; Tashiro, K.; Hatano, T.; Oishi, Y. [a clinical study of secondary osteoporosis induced by endocrine therapy for prostate cancer]. Nihon Hinyokika Gakkai Zasshi 2000, 91, 573-578.
9. Galvao, D.A.; Spry, N.A.; Taaffe, D.R.; Newton, R.U.; Stanley, J.; Shannon, T.; Rowling, C.; Prince, R. Changes in muscle, fat and bone mass after 36 weeks of maximal androgen blockade for prostate cancer. BJU Int. 2008, 102, 44-47.
10. Greenspan, S.L.; Coates, P.; Sereika, S.M.; Nelson, J.B.; Trump, D.L.; Resnick, N.M. Bone loss after initiation of androgen deprivation therapy in patients with prostate cancer. J. Clin. Endocrinol. Metab. 2005, 90, 6410-6417.
11. Higano, C.; Shields, A.; Wood, N.; Brown, J.; Tangen, C. Bone mineral density in patients with prostate cancer without bone metastases treated with intermittent androgen suppression. Urology 2004, 64, 1182-1186.
12. Isahaya, E.; Hara, N.; Nishiyama, T.; Hoshii, T.; Takizawa, I.; Takahashi, K. Bone metabolic disorder in patientswith prostate cancer receiving androgen deprivationtherapy (adt): Impact of adton the growth hormone/ insulin-like growth factor-1/parathyroid hormoneaxis. Prostate 2010, 70, 155-161.
13. Kato, S.; Kawase, M.; Kato, D.; Ishida, T.; Uno, M.; Fujimoto, Y.; Masue, T.; Masue, N.; Deguchi, T. Decrease of bone mineral density in japanese patients with non-metastatic prostate cancer treated with androgen deprivation therapy. J. Bone Miner. Metab. 2018,doi:10.1007/s00774-017-0897-5.
14. Kim, S.H.; Joung, J.Y.; Kim, S.; Rha, K.H.; Kim, H.G.; Kwak, C.; Lee, J.Y.; Jeon, S.S.; Hong, S.K.; Jeong, H. et al. Comparison of bone mineral loss by combined androgen block agonist versus gnrh in patients with prostate cancer: A 12 month-prospective observational study. Sci. Rep. 2017, 7, 39562.
15. Kim, S.H.; Seong do, H.; Yoon, S.M.; Choi, Y.D.; Song, Y.; Song, H.; Choi, E. Bone health and its correlates in korean prostate cancer patients receiving androgen deprivation therapy. Eur. J. Oncol. Nurs. 2016, 21, 197-204.
16. Kimura, T.; Koike, Y.; Aikawa, K.; Kimura, S.; Mori, K.; Sasaki, H.; Miki, K.; Watanabe, K.; Saito, M.; Egawa, S. Mp87-13 androgen deprivation therapy causes vitamin k deficiency and decreased bone mineral density in castration-sensitive prostate cancer: A prospective study. J. Urol 2018, 199, e1192-e1193.
17. Kiratli, B.J.; Srinivas, S.; Perkash, I.; Terris, M.K. Progressive decrease in bone density over 10 years of androgen deprivation therapy in patients with prostate cancer. Urology 2001, 57, 127-132.
18. Langley, R.E.; Kynaston, H.G.; Alhasso, A.A.; Duong, T.; Paez, E.M.; Jovic, G.; Scrase, C.D.; Robertson, A.; Cafferty, F.; Welland, A. et al. A randomised comparison evaluating changes in bone mineral density in advanced prostate cancer: Luteinising hormone-releasing hormone agonists versus transdermal oestradiol. Eur. Urol. 2016, 69, 1016-1025.
19. Lee, H.; McGovern, K.; Finkelstein, J.S.; Smith, M.R. Changes in bone mineral density and body composition during initial and long-term gonadotropin-releasing hormone agonist treatment for prostate carcinoma. Cancer 2005, 104, 1633-1637.
20. Maillefert, J.F.; Sibilia, J.; Michel, F.; Saussine, C.; Javier, R.M.; Tavernier, C. Bone mineral density in men treated with synthetic gonadotropin-releasing hormone agonists for prostatic carcinoma. J. Urol 1999, 161, 1219-1222.
21. Mittan, D.; Lee, S.; Miller, E.; Perez, R.C.; Basler, J.W.; Bruder, J.M. Bone loss following hypogonadism in men with prostate cancer treated with gnrh analogs. J. Clin. Endocrinol. Metab. 2002, 87, 3656-3661.

22. Miyaji, Y.; Saika, T.; Yamamoto, Y.; Kusaka, N.; Arata, R.; Ebara, S.; Nasu, Y.; Tsushima, T.; Kumon, H. Effects of gonadotropin-releasing hormone agonists on bone metabolism markers and bone mineral density in patients with prostate cancer. Urology 2004, 64, 128-131.
23. Miyazawa, Y.; Sekine, Y.; Syuto, T.; Nomura, M.; Koike, H.; Matsui, H.; Shibata, Y.; Ito, K.; Suzuki, K. Evaluation of bone turnover / quality markers and bone mineral density in prostate cancer patients receiving androgen deprivation therapy with or without denosumab. Anticancer Res. 2017, 37, 3667-3671.
24. Miyazawa, Y.; Sekine, Y.; Syuto, T.; Nomura, M.; Koike, H.; Matsui, H.; Shibata, Y.; Ito, K.; Suzuki, K. Effect of androgen-deprivation therapy on bone mineral density in japanese patients with prostate cancer. In Vivo 2018, 32, 409-412.
25. Morote, J.; Martinez, E.; Trilla, E.; Esquena, S.; Abascal, J.M.; Encabo, G.; Reventos, J. Osteoporosis during continuous androgen deprivation: Influence of the modality and length of treatment. Eur. Urol. 2003, 44, 661-665.
26. Ojeda-Bruno, S.; Naranjo-Hernández, A.; Lloret, M.; Domínguez-Cabrera, C.; Francisco-Hernández, F.; Lara, P.C. Loss of bone mass in androgen-deprivation adjuvant hormonal therapy in high risk localized protate cancer patients. Radiother. Oncol. 2012, 103, S46-S47.
27. Saad, F.; Feldman, R.; Heracek, J.; Ke, C.; Goessl, C. Natural history of bone loss in men with prostate cancer receiving androgen-deprivation therapy. J. Urol 2009, 181, 593-594.
28. Smith, M.R.; McGovern, F.J.; Fallon, M.A.; Schoenfeld, D.; Kantoff, P.W.; Finkelstein, J.S. Low bone mineral density in hormone-naïve men with prostate carcinoma. Cancer 2001, 91, 2238-2245.
29. Stoch, S.A.; Parker, R.A.; Chen, L.; Bubley, G.; Ko, Y.J.; Vincelette, A.; Greenspan, S.L. Bone loss in men with prostate cancer treated with gonadotropin-releasing hormone agonists. J. Clin. Endocrinol. Metab. 2001, 86, 2787-2791.
30. Suzuki, Y.; Aikawa, K.; Oishi, Y.; Yamazaki, H.; Onishi, T.; Suzuki, M.; Kobari, T.; Endo, K.; Yanada, S.; Kato, N. et al. [a clinical study of decreased bone density in the patients treated with long-term luteinizing hormone releasing hormone analogue (lhrh-a)--the risk of iatrogenic osteoporosis due to treatment of carcinoma of prostate]. Nihon Hinyokika Gakkai Zasshi 1998, 89, 961-966.
31. Wang, A.; Karunasinghe, N.; Plank, L.; Zhu, S.; Osborne, S.; Bishop, K.; Brown, C.; Schwass, T.; Masters, J.; Holmes, M. et al. Effect of androgen deprivation therapy on bone mineral density in a prostate cancer cohort in new zealand: A pilot study. Clin. Med. Insights Oncol. 2017, 11, 1179554917733449.
32. Wang, W.; Yuasa, T.; Tsuchiya, N.; Maita, S.; Kumazawa, T.; Inoue, T.; Saito, M.; Ma, Z.; Obara, T.; Tsuruta, H. et al. Bone mineral density in japanese prostate cancer patients under androgen-deprivation therapy. Endocr. Relat. Cancer 2008, 15, 943-952.
33. Wei, J.T.; Gross, M.; Jaffe, C.A.; Gravlin, K.; Lahaie, M.; Faerber, G.J.; Cooney, K.A. Androgen deprivation therapy for prostate cancer results in significant loss of bone density. Urology 1999, 54, 607-611.
34. Yu, E.Y.; Kuo, K.; Gulati, R.; Jiang, P.Y.; Higano, C.S. Dynamics of bone mineral density during intermittent androgen deprivation for men with biochemical recurrence of prostate cancer. J. Clin. Oncol. 2010, 28.
35. Ziaran, S.; Goncalves, F.M.; Wendl, J.; Trebaticky, B.; Breza, J.S. Evaluation of bone mass density on patients with prostate cancer prior to the start of androgen deprivation therapy. Bratisl. Lek. Listy 2009, 110, 559-562.

Appendix A.3. PRISMA checklist

Section/topic	#	Checklist item	Reported on page #
**TITLE**			
Title	1	Identify the report as a systematic review, meta-analysis, or both.	1
**ABSTRACT**			
Structured summary	2	Provide a structured summary including, as applicable: background; objectives; data sources; study eligibility criteria, participants, and interventions; study appraisal and synthesis methods; results; limitations; conclusions and implications of key findings; systematic review registration number.	1
**INTRODUCTION**			
Rationale	3	Describe the rationale for the review in the context of what is already known.	1,2
Objectives	4	Provide an explicit statement of questions being addressed with reference to participants, interventions, comparisons, outcomes, and study design (PICOS).	2
**METHODS**			
Protocol and registration	5	Indicate if a review protocol exists, if and where it can be accessed (e.g., Web address), and, if available, provide registration information including registration number.	2
Eligibility criteria	6	Specify study characteristics (e.g., PICOS, length of follow-up) and report characteristics (e.g., years considered, language, publication status) used as criteria for eligibility, giving rationale.	2,3
Information sources	7	Describe all information sources (e.g., databases with dates of coverage, contact with study authors to identify additional studies) in the search and date last searched.	2
Search	8	Present full electronic search strategy for at least one database, including any limits used, such that it could be repeated.	2
Study selection	9	State the process for selecting studies (i.e., screening, eligibility, included in systematic review, and, if applicable, included in the meta-analysis).	2,3
Data collection process	10	Describe method of data extraction from reports (e.g., piloted forms, independently, in duplicate) and any processes for obtaining and confirming data from investigators.	3
Data items	11	List and define all variables for which data were sought (e.g., PICOS, funding sources) and any assumptions and simplifications made.	2
Risk of bias in individual studies	12	Describe methods used for assessing risk of bias of individual studies (including specification of whether this was done at the study or outcome level), and how this information is to be used in any data synthesis.	3
Summary measures	13	State the principal summary measures (e.g., risk ratio, difference in means).	3
Synthesis of results	14	Describe the methods of handling data and combining results of studies, if done, including measures of consistency (e.g., I^2) for each meta-analysis.	3

Section/topic	#	Checklist item	Reported on page #
Risk of bias across studies	15	Specify any assessment of risk of bias that may affect the cumulative evidence (e.g., publication bias, selective reporting within studies).	3
Additional analyses	16	Describe methods of additional analyses (e.g., sensitivity or subgroup analyses, meta-regression), if done, indicating which were pre-specified.	3
**RESULTS**			
Study selection	17	Give numbers of studies screened, assessed for eligibility, and included in the review, with reasons for exclusions at each stage, ideally with a flow diagram.	3, figure 1
Study characteristics	18	For each study, present characteristics for which data were extracted (e.g., study size, PICOS, follow-up period) and provide the citations.	3, table 1
Risk of bias within studies	19	Present data on risk of bias of each study and, if available, any outcome level assessment (see item 12).	Table 2
Results of individual studies	20	For all outcomes considered (benefits or harms), present, for each study: (a) simple summary data for each intervention group (b) effect estimates and confidence intervals, ideally with a forest plot.	Table 1
Synthesis of results	21	Present results of each meta-analysis done, including confidence intervals and measures of consistency.	4
Risk of bias across studies	22	Present results of any assessment of risk of bias across studies (see Item 15).	4, Table 2
Additional analysis	23	Give results of additional analyses, if done (e.g., sensitivity or subgroup analyses, meta-regression [see Item 16]).	Figure 2
**DISCUSSION**			
Summary of evidence	24	Summarize the main findings including the strength of evidence for each main outcome; consider their relevance to key groups (e.g., healthcare providers, users, and policy makers).	8,9
Limitations	25	Discuss limitations at study and outcome level (e.g., risk of bias), and at review-level (e.g., incomplete retrieval of identified research, reporting bias).	10
Conclusions	26	Provide a general interpretation of the results in the context of other evidence, and implications for future research.	10
**FUNDING**			
Funding	27	Describe sources of funding for the systematic review and other support (e.g., supply of data); role of funders for the systematic review.	10

From: Moher D, Liberati A, Tetzlaff J, Altman DG, The PRISMA Group (2009). Preferred Reporting Items for Systematic Reviews and Meta-Analyses: The PRISMA Statement. PLoS Med 6(7): e1000097. doi:10.1371/journal.pmed1000097
